# A Front-Side Microfabricated Thermoresistive Gas Flow Sensor for High-Performance, Low-Cost and High-Yield Volume Production

**DOI:** 10.3390/mi11020205

**Published:** 2020-02-17

**Authors:** Dan Xue, Jiachou Wang, Xinxin Li

**Affiliations:** 1 State Key Laboratory of Transducer Technology, Shanghai Institute of Microsystem and Information Technology, Chinese Academy of Sciences, Shanghai 200050, China; xuedan221@mail.sim.ac.cn; 2 Center of Materials Science and Optoelectronics Engineering, University of Chinese Academy of Sciences, Beijing 100049, China

**Keywords:** gas flow sensor, single-side bulk micromachining, ultrahigh sensitivity

## Abstract

In this paper, we present a novel thermoresistive gas flow sensor with a high-yield and low-cost volume production by using front-side microfabricated technology. To best improve the thermal resistance, a micro-air-trench between the heater and the thermistors was opened to minimize the heat loss from the heater to the silicon substrate. Two types of gas flow sensors were designed with the optimal thermal-insulation configuration and fabricated by a single-wafer-based single-side process in (111) wafers, where the type A sensor has two thermistors while the type B sensor has four. Chip dimensions of both sensors are as small as 0.7 mm × 0.7 mm and the sensors achieve a short response time of 1.5 ms. Furthermore, without using any amplification, the normalized sensitivity of type A and type B sensors is 1.9 mV/(SLM)/mW and 3.9 mV/(SLM)/mW for nitrogen gas flow and the minimum detectable flow rate is estimated at about 0.53 and 0.26 standard cubic centimeter per minute (sccm), respectively.

## 1. Introduction

Gas flow is a necessary parameter in numerous industrial and laboratory on-chip applications [1,2,3]. Due to the development of microelectronic manufacturing processes, the miniaturization of instruments has attracted considerable attention from researchers, which has led to the development of silicon-based gas flow sensors along the direction of more miniaturization, lower cost and higher performance [4,5]. Silicon-based gas flow sensors are mainly divided into thermal and nonthermal types. It should be noted that thermal flow sensors do not require any moving parts [6]. Thus, they could be implemented in Complementary Metal-Oxide-Semiconductor (CMOS) processes as the easiest flow measurement devices due to their structural simplicity. According to the working principle of the sensitive element, thermal gas flow sensors can be divided into time-of-flight, calorimetric and hot-wire/hot-film types [7]. Among the sensors, a thermoresistive microcalorimetric flow sensor calibrates the gas flow by detecting the temperature difference of the upstream and downstream thermistors, which difference is caused by the flow transport of the gas [8]. Furthermore, the thermoresistive microcalorimetric flow sensor is widely used for its low power consumption and high detection accuracy.

To satisfy the rapid development of automotive electronics, biomedical instruments, process control systems and so on, easy miniaturization and low-cost volume production of the silicon-based thermoresistive gas flow sensors are needed. However, conventional thermoresistive flow sensors are generally fabricated using double-sided micromachining [9]. With the technical approaches, thermal insulation membrane is formed by potassium hydroxide (KOH) anisotropic etching from the wafer backside. The anisotropic etching-induced inclined sidewalls cause the chip size to be quite large and the back-sided KOH etching is time-consuming, which yield a higher batch-fabrication cost [10]. Thus, developing a new strategy to minimize the chip size for realizing batch-fabrication with a lower cost is an important issue. Recently, a commercial 0.35 µm 2P4M microelectromechanical systems (MEMS) process was used in [11] to fabricate a sensor from the front-side of a (100) wafer. The approach decreased the chip-size and fabrication cost. However, the insulation membrane was released through XeF_2_ isotropic etching, Thus, a depth-limited insulation cavity was formed, which would increase the heat loss, lower the thermal resistance and cause a relatively lower sensitivity of the sensor. Here, this paper expands on preliminary research presented in [12] and explores a tiny-sized ultrasensitive thermoresistive gas flow sensor that is single-side processed in an ordinary single-polished (111) silicon wafer. By changing the number of thermoresistive sensors, we developed two types of differential thermoresistive gas flow sensors, a half-bridge type and a full-bridge type.

## 2. Sensor Design

**Figure 1:**
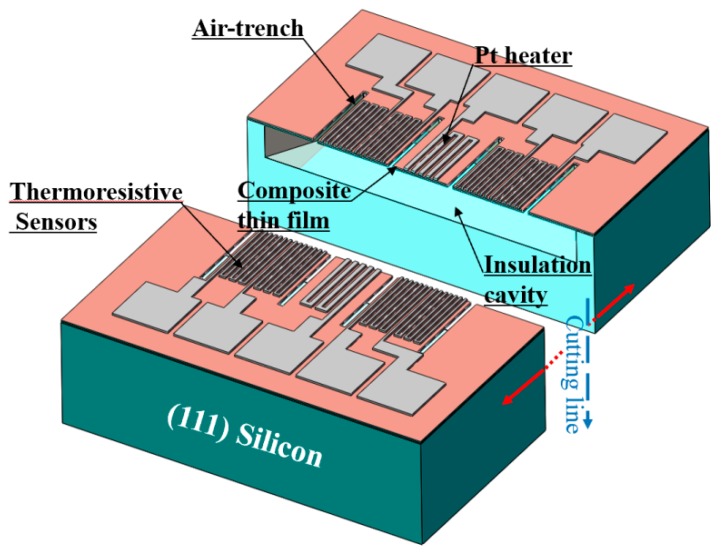
Three-dimensional schematic of the type B gas flow sensor on a single (111) wafer.

Two types of single-side processed micro gas flow sensors were fabricated in the (111) silicon wafer. Type A and type B flow sensors have the same structure and configuration, except for the number of the thermistors. Herein, the type A sensor has two thermistors while the type B sensor has four thermistors. Figure 1 shows the three-dimensional schematic of our proposed type B gas flow sensor. A Pt heater was positioned at the center of the Si_3_N_4_/SiO_2_ membrane and each of the two Pt thermistors was deposited symmetrically on either side of the heater. Air-trench between them was further opened to increase the thermal resistance, which is helpful to minimize the heat loss from the heater to the substrate. Additionally, the thermistors establish a detection circuit to detect changes in the flow rate of the gas. Moreover, enhanced insulation of the suspension film and silicon substrate was realized by introducing a 50 μm-deep isolation cavity underneath the Si_3_N_4_/SiO_2_ membrane, leading to facilitated heat exchange between gas flow and the sensor.

**Figure 2:**
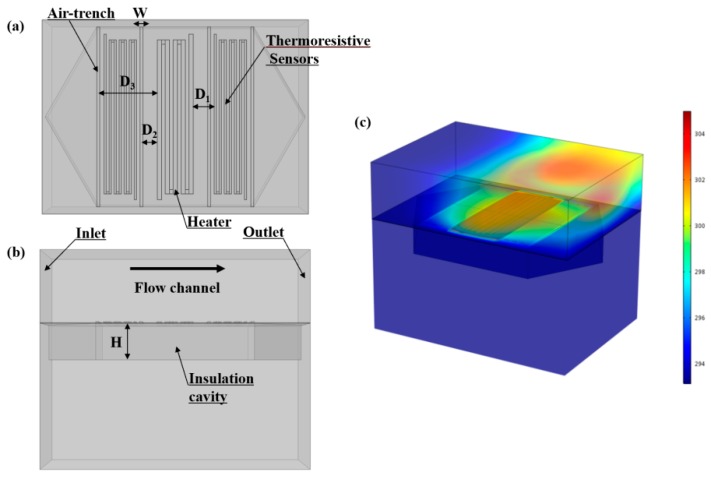
Three-dimensional simulation model of the gas flow sensor. (**a**) Top view of simulation model (D_1_, the distance between the thermistors and the heater; W, the width of the air-trench; D_2_ or D_3_, the distance between the air-trench and the heater). (**b**) Front view of simulation model (H, the depth of the thermal isolation cavity). (**c**) Temperature distribution diagram of the flow sensor.

A finite element model of gas flow sensors was developed by COMSOL Multiphysics (V5.3, COMSOL Inc, Burlington, MA, USA), which was employed to analyze and optimize the structure of the designed sensors for minimizing the heat loss. Figure 2a,b show the 3D models of the flow sensor based on a 1:1 structure size, and Figure 2c shows the temperature distribution of the flow sensor at a flow rate of 1.0 m/s and a heater power of 4 mW. The simulation coupled the heat transfer effects between the laminar flow and the solid. The model included two physical fields: the laminar flow field and the temperature field. For laminar flow field, the inlet and outlet of the flow channel were established and other boundary conditions defined as no-slip. For temperature field, the solid heat transfer was coupled with flow heat transfer, and the domain of the two heat transfer modes was configured in the flow sensor. The power of the heater was set with the heat consumption rate. The temperature of the inlet was assigned to 293 K and other boundaries in the model were identified using heat flux to describe the effects of heat convection.

**Figure 3:**
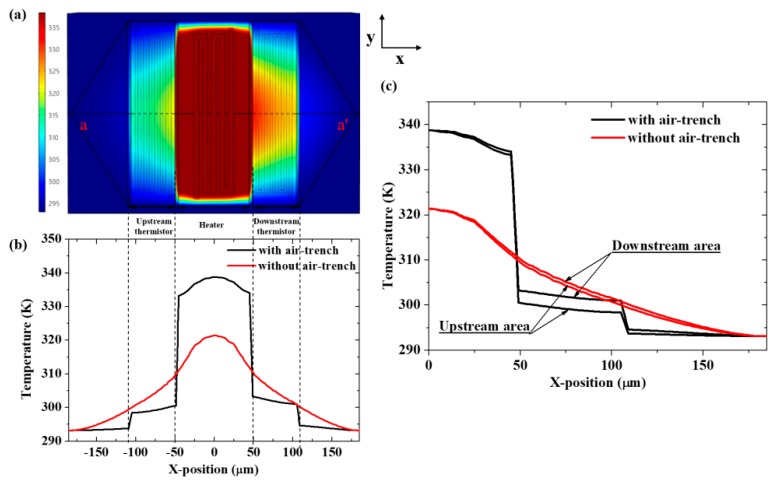
Simulation result of the gas flow sensor with air-trench and without air-trench. (**a**) Temperature distribution diagram of the composite thin film. (**b**) Composite thin film temperature distribution diagram of simulation model. (**c**) Composite thin film temperature distribution at the upstream and downstream symmetrical position.

Figure 3a shows the COMSOL-simulated temperature distribution on the upper surface of the dielectric composite membrane with a nitrogen gas flow rate of 1.0 m/s and a heater power of 4 mW. Figure 3b shows the temperature distribution on the top surface of the dielectric film along the straight-line a–a’ in Figure 3a. The temperature in the Pt heater area of the flow sensor with air-trench is higher than that of the flow sensor without air-trench, while the temperature in the thermistor area is lower under the same condition. It is mainly due to the fact that the thermal resistance of the air-trench is larger than that of the dielectric composite film, resulting in a smaller heat loss of the sensor with air-trench than that of the sensor without air-trench. Figure 3c exhibits the temperature distribution upstream and downstream from the gas flow sensor. Obviously, the gas flow sensor with air-trench has a higher temperature difference (∆T) between the upstream thermistor and the downstream thermistor than the traditional flow sensor without air-trench. According to ∆R = α × R × ∆T (where ∆R is the resistance difference between the upstream Pt thermistor and downstream Pt thermistor, R is the resistance of the Pt thermistor at a zero-flow rate and α is the Pt temperature coefficient), the higher the ∆T, the bigger the ∆R. Therefore, the proposed gas flow sensor with air-trench has a higher sensitivity.

**Figure 4:**
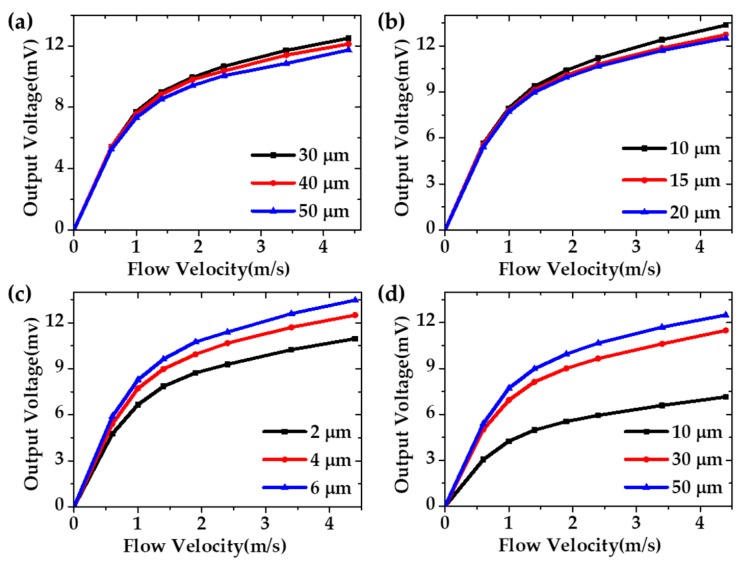
The simulation results of voltage and flow velocity with different structure parameters. (**a**) Distance between the thermistors and the heater (D_1_). (**b**) Distance between the air-trench and the heater (D_2_). (**c**) Width of the air-trench (W). (**d**) Depth of the thermal isolation cavity (H).

In addition, simulation analysis was used to optimize the structural parameters to obtain high performance of the sensor. Figure 4 presents the simulation results of output voltage versus flow velocity of the gas flow sensor with different structural parameters (since the above parameters have similar influence on the temperature distribution of type A and type B gas flow sensors, only the simulation results of type A are discussed). As shown in Figure 4a, the distance between the thermistors and the heater (D_1_) was set to 30, 40 and 50 µm. It was observed that the output voltage decreased along with increasing D_1_. From Figure 4b, it can be seen that the output voltage decreased when the distance between the air-trench and the heater (D_2_) increased from 10 to 20 µm. Furthermore, as shown in Figure 4c,d, the width of the air- trench (W) was set to 2, 4 and 6 µm, while the depth of the thermal isolation cavity (H) was 10, 30 and 50 µm. It was found that the output voltage had a positive relationship with W and H. Thus, by comparing the simulation results, higher H and W with lower D_1_ and D_2_ yielded higher output voltage, which indicates a higher sensitivity of the flow sensor.

Combining this with the simulation results and MIS (micro-openings interetch and sealing) micromachining processing [13], the detailed structural parameters are listed in Table 1.

## 3. Fabrication

**Figure 5:**
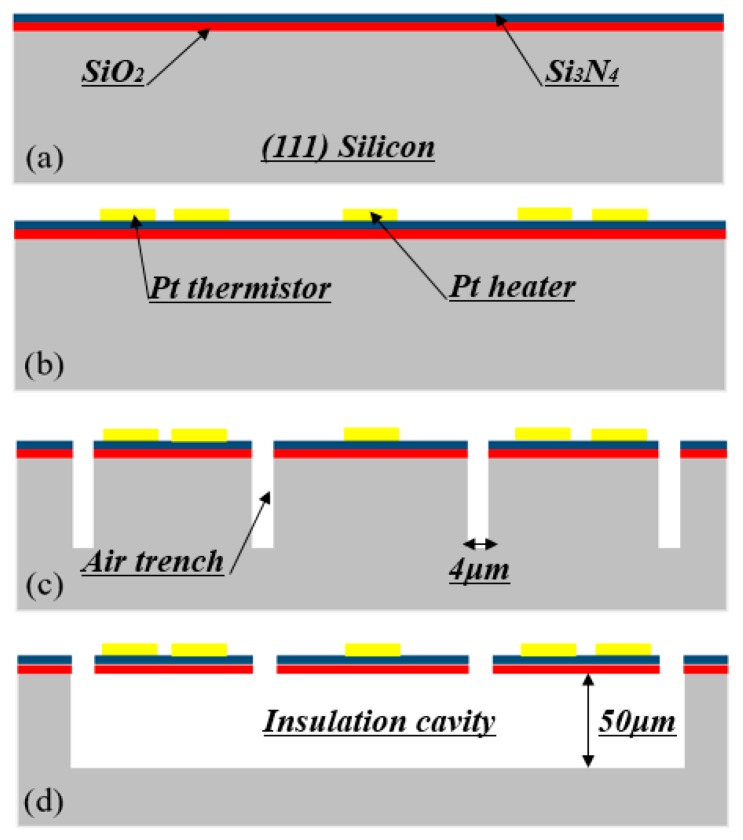
Detailed fabrication steps of the proposed two types of thermoresistive gas flow sensor.

To fabricate the proposed gas flow sensors, only two mask layers were provided; this much simpler process than previously reported promoted device yield and a remarkably lower cost. As displayed in Figure 5, the whole process was always carried out at the front side of (111) silicon. Starting from an n-type four inch (111) single-polished silicon wafer in the resistance of 3~10 Ω·cm, the detailed fabrication steps are described as follows:

(a) Firstly, a 0.2 µm-thick SiO_2_ layer was grown by thermal oxidiation, then low-pressure chemical vapor deposition (LPCVD) was employed to form a 0.8 µm-thick low-stress SiN layer on top of the silicon dioxide layer.

(b) The first photolithography was used to form the pattern of the thermistors and heaters after a Ti–W/Pt layer (50 nm/500 nm in thickness) was sputtered on top of the low-stress Si_3_N_4_ layer. The type A sensors pattern with two thermistors, type B sensors pattern with four thermistors. With the photoresist as an etching mask, the Ti–W/Pt layer was etched by ion beam etching equipment.

(c) The second photolithography was conducted to pattern the four air trenches along <211>-orientation. Reactive-ion etching (RIE) was used to etch off the Si_3_N_4_/SiO_2_ membrane to expose bare silicon and deep-RIE was processed to deepen the trenches for defining the height of the insulation cavity with the patterned photoresist layer as a mask.

(d) Finally, the wafer was dipped into 25% aqueous tetramethylammonium hydroxide (TMAH) under 80 °C for about 1.0 h to complete the bottom release by lateral underetching and form the suspension for the Si_3_N_4_/SiO_2_ membrane. Without protection of the passivation layer, the trench undergoes etching along the <110> and <211> orientation. The hexagonal-shaped insulation cavity was excavated with all the etching-stop boundaries as {111} planes.

**Figure 6:**
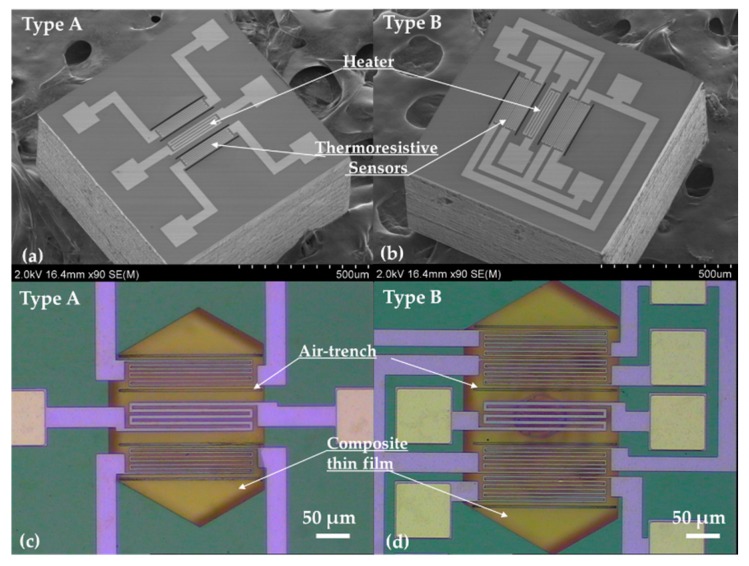
The micrographs showing the fabricated gas flow sensor. (**a**,**b**) SEM showing the gas flow sensor. (**c**,**d**) Top-view micrograph image of the gas flow sensor.

Figure 6 presents the two types of as-fabricated gas flow sensors, where the suspended Si_3_N_4_/SiO_2_ membrane, Pt heater, thermistor and air-trench can be easily observed. Further, the top-view optical micrograph (OM) images of the whole fabricated sensors presented in Figure 6c,d confirm the intact composite membrane. In addition, the scanning electron microscope (SEM) images in Figure 6a,b clarify that the size of sensor chips shrunk to 0.7 mm × 0.7 mm for both sensors.

## 4. Device Characterization

**Figure 7:**
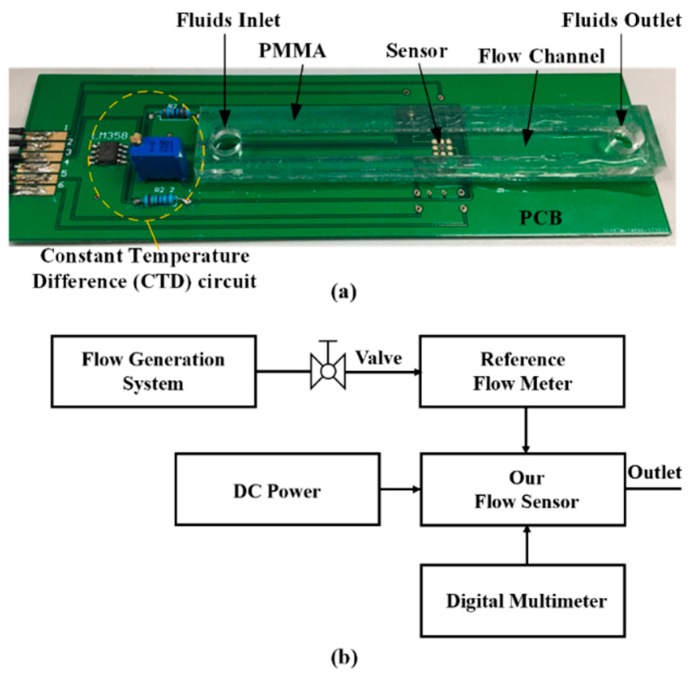
(**a**) Packaged gas flow sensor in a poly(methyl methacrylate) (PMMA) flow channel. (**b**) Flow diagram illustrating the experimental setup for testing the sensor.

To investigate the performance of the sensor, as shown in Figure 7a, the sensor was directly glued on the surface of a PCB board and then encapsulated in a poly(methyl methacrylate) (PMMA) flow channel with a 5.5 mm × 1.5 mm cross section. The packaged sensors were tested under nitrogen flow. The schematic of the test flow is displayed in Figure 7b. The nitrogen tank was used as the gas source, and a commercial flow sensor, Molecular Analysis Series 8000S (Molecular Analysis LLC, Wilmington, DE, USA), was used as a reference flow meter.

**Figure 8:**
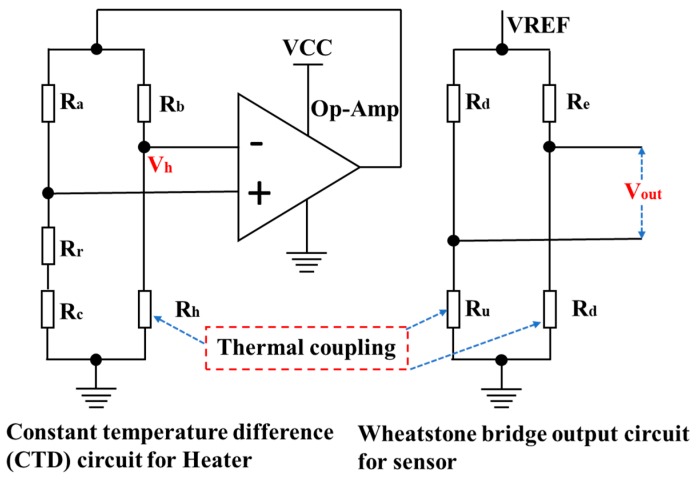
The constant temperature difference circuit and the Wheatstone bridge output circuit for the flow sensor.

A constant temperature difference (CTD) circuit efficiently compensates for output drifting, which is caused by the fluctuation of ambient temperature. As displayed in Figure 8, the circuit is formed with the amplifier LM358 (Risym, Shenzhen, China), the heater R_h_, the on-chip reference ambient temperature sensor R_r_, the compensation resistor R_c_, resistor R_a_ and resistor R_b_. The operating principle of this CTD circuit is described in [14]. Our design incorporates the Wheatstone bridge readout method [15], for the type A sensor, the circuit is constituted with upstream and downstream resistors with the addition of two on-chip resistors. For the type B sensor, the Wheatstone bridge circuit is achieved only with upstream and downstream resistors. DC power (Agilent E3631A, Keysight Technologies, Santa Rosa, CA, USA) is supplied on the CTD mode and Wheatstone bridge of the sensor. Without any amplification, the output signals of the thermoresistive sensor are read out by a digital multimeter (Agilent 34410A, Keysight Technologies).

**Figure 9:**
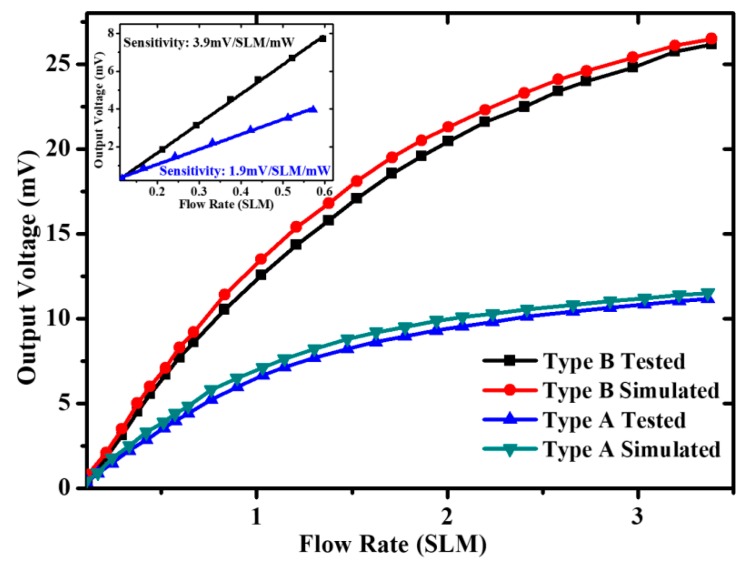
Measured output voltage of the fabricated sensor as a function of input flow rate.

Within a nitrogen gas flow range of 0–3.4 SLM (0~5.0 m/s), the measured output voltage of the fabricated sensor versus the applied nitrogen gas flow is depicted in Figure 9. The black symbols and blue symbols represent the results of an actual measurement of the type A and type B sensors. With respect to the input heating power, the ultrahigh normalized sensitivity of the type A and type B sensors determined in low flow region are 1.9 mV/SLM/mW (1.26 mV/(m/s)/mW) and 3.9 mV/SLM/mW (2.59 mV/(m/s)/mW). The red symbols and green symbols indicate the results from the COMSOL simulation. Obviously, the tested results are slightly lower than the theoretical results. The discrepancy is mainly due to the fabrication imperfections. For example, the depth of the thermal isolation cavity may be smaller than the design value after deep-RIE, causing more heat loss. Therefore, the output voltage may be smaller at a given heating power.

As listed in Table 2, the sensitivity of both sensors is one order of magnitude higher than the reported thermoresistive microcalorimetric gas flow sensor.

**Figure 10:**
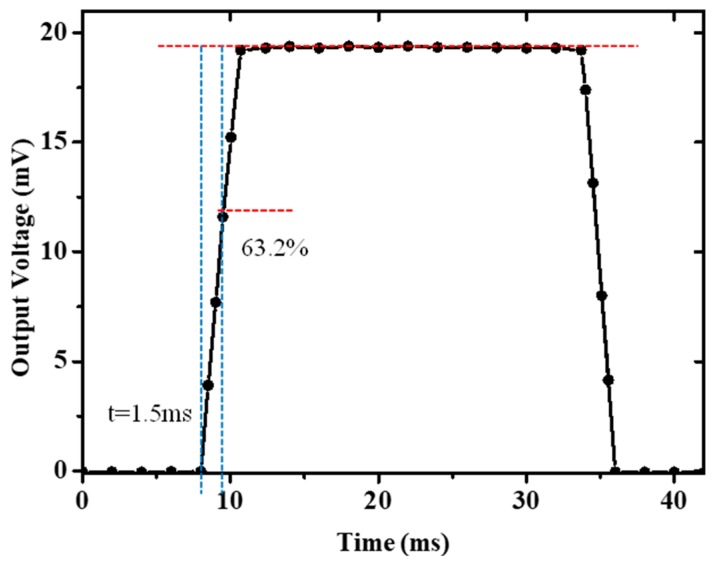
Dynamical response of the type B sensor to Pt heater with a heating power step input.

Because the thermistors of the two sensors have the same resistance value, the step response and noise of the two sensors keep the same value. According to the definition of the response time of the flow system [16], with a constant input nitrogen gas flow of 1.8 SLM, an electric impulse heating power of 4.0 mW was applied to the heater directly to estimate the dynamical response of the type B flow sensor. As shown in Figure 10, the sensor exhibits a response time of 1.5 ms.

**Figure 11:**
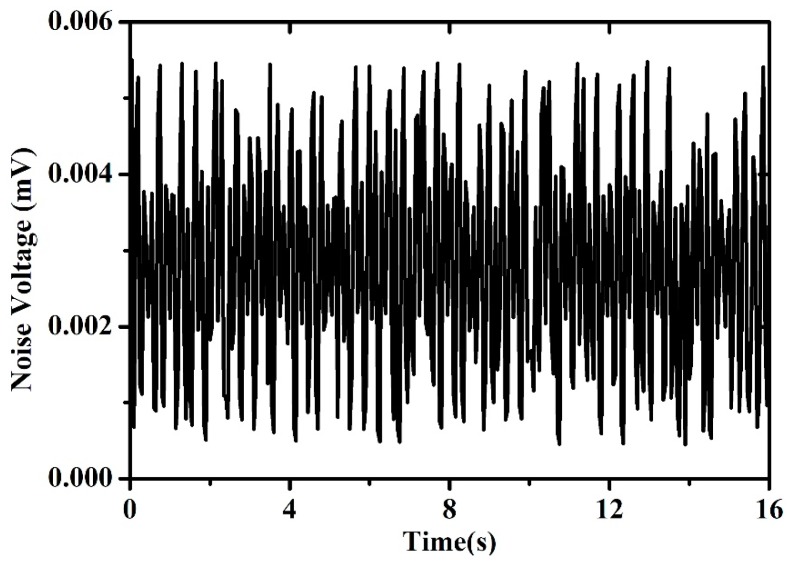
Output noise of the type B sensor plotted as a function of time in the zero-flow case.

Figure 11 shows a time diagram of the output noise of the type B flow sensor measured over intervals of 16 s in condition of zero-flow rate. The standard deviation of the noise voltage is estimated at nearly 0.002 mV. Considering the sensitivity, the minimum detectable flow rate [17] of the type A and type B sensors are 0.53 and 0.26 sccm, respectively.

## 5. Conclusions

A thermoresistive gas flow sensor was fabricated with a single wafer-based single-side process in (111) wafers. The simplified fabrication process facilitated an ultrasmall chip size of 0.7 × 0.7 mm volume manufacturing. Additionally, the designed insulation membrane with air-trench effectively reduced and minimized heat loss from the heater to the substrate. The type A and the type B gas flow sensors achieved a remarkable normalized sensitivity of 1.9 mV/SLM/mW (1.26 mV/(m/s)/mW) and 3.9 mV/SLM/mW (2.59 mV/(m/s)/mW), respectively, within a nitrogen gas flow ranging from 0 to 3.4 SLM (from 0 to 5.0 m/s). Therefore, the high-performance, low-cost and high-yield volume production gas flow sensors would satisfy practical applications.

## Figures and Tables

**Table 1:** Detailed dimensions for the gas flow sensor.

Geometric Parameters	Half-Bridge	Full-Bridge
Depth of the thermal isolation cavity (H)	50 µm	50 µm
Width of the air-trench (W)	4 µm	4 µm
Distance between thermistors and heater (D_1_)	30 µm	30 µm
Distance between air-trench and heater (D_2_, D_3_)	20 µm, 80 µm	20 µm, 130 µm

**Table 2:** Summary of the previous thermoresistive calorimetric flow sensors and our work.

Ref.	Area mm^2^	Thin Film	Structure/Fabrication Style	Sensitivity* mV/(m/s)/mW
[9]	36	SiO_2_	Double-sided process	0.039
[11]/[14]	2.25	SiO_2_	Single-sided process	0.154
[16]	3.4	Si/SiO_2_	Silicon-On-Insulator (SOI)-wafer/single-sided process	0.112
Our work	0.49	SiN/ SiO_2_	Single-polished single-wafer/ single-sided process	1.26/2.59
